# Dynamic molecular choreography of circadian rhythm disorders (DMCRD): a prospective cohort study protocol

**DOI:** 10.1186/s12883-022-02799-8

**Published:** 2022-07-21

**Authors:** Xiaoli Chen, Dongze Li, Yu Jia, Yanmei Liu, Yan Ma, Rui Zeng, Zhi Wan, Lei Ye

**Affiliations:** 1 Emergency Department of West China Hospital, Sichuan University/West China School of Nursing, Sichuan University, 37 Guoxue Road, Chengdu, 610041 Sichuan China; 2 Institute of Disaster Medicine, Sichuan University, Chengdu, China; 3 Chinese Evidence-Based Medicine Center, West China Hospital, Sichuan University, Chengdu, China; 4 School of Public Health, Xinjiang Medical University, Urumqi, China; 5 Department of Cardiology, West China Hospital, Sichuan University, Chengdu, China

**Keywords:** Circadian rhythm disorders, Molecular mechanism, Biological rhythm, Health assessment

## Abstract

**Background:**

Circadian rhythm disorders (CRDs) are closely associated with the occurrence and development of various diseases, such as inflammatory and cardiovascular diseases, as well as tumors. The impact of a CRD on bodily health is a complex and comprehensive process, and its molecular mechanisms and signaling pathways are still unclear. We therefore aimed to investigate the molecular mechanism variation and adverse outcomes associated with CRDs in a prospective cohort of CRD cases and controls at term using multiomics data. The study has been tasked with developing a precise health promotion model for the prevention and management of CRDs.

**Methods:**

This will be a 5-year prospective cohort study centered on the health management of individuals with CRDs. One hundred volunteers were recruited and had undergone baseline specimen collection, health examination, and health assessment. All of them will be followed up every year using the same protocol, and their biological specimens will be subjected to multiomics analysis after standardized processing.

**Discussion:**

Longitudinal health examination, health assessment, and multiomics data will be analyzed to study the impact of CRDs on the volunteers’ health status. The results of this study will promote the development of targeted health management programs based on precision medicine.

**Trial registration:**

The clinical study registration has been completed (Trial Registration No. ChiCTR2100047242).

## Background

A biological rhythm is a consistent and regular pattern of changes in bodily activities. It involves the whole process from gene transcription to daily behaviors, and it regulates various metabolic and physiological functions of the human body [1]. The balance and stability of biological rhythms can ensure healthy life activities. However, social progress, an accelerated pace of life, and increasing social pressure lead to an unhealthy lifestyle, which may include long-term staying up late, sleep deprivation, as well as reversal of day and night sleep patterns.

Biological rhythm disorders are becoming prevalent. Large-scale experimental studies have found that long-term circadian rhythm disorders (CRDs) are associated with an imbalance in food intake, body weight, and energy expenditure. CRDs can also cause metabolic syndrome, cardiovascular disease, and glucose homeostasis [2–4]. A fair amount of evidence has shown that the disturbance of circadian genes contributes to the occurrence and progression of multisystem diseases (e.g., circulatory system, immune system, nervous system, and endocrine system diseases) and tumors [5–7]. In a simulation of night and day shifts, the Washington State University found that staying up late disturbed the natural rhythm of cancer-related genes and eventually led to reduced DNA repair efficiency and increased cancer risk during the DNA repairing process [8]. Rogulja et al. reported that sleep deprivation induced the accumulation of reactive oxygen species in the intestine, which could, in turn, lead to oxidative stress and increase the risk of death [9].

On the other hand, the molecular mechanisms of the effects of circadian rhythm disturbance on bodily health are still unclear. Previous molecular studies had restricted breadth, employed limited biological processes, and used only a single level of analysis to reveal molecular data related to CRDs. Few studies have investigated the effects of bodily health on molecules and signaling pathways. Data on the mechanisms by which CRDs affect physical health thus remain incomprehensive.

Multiomics meta-analysis refers to the normalization processing of different data groups, such as transcriptomics, proteomics, metabolomics, and peptidomics data. It entails comparative analysis followed by the integration of multiomics data on biological processes, from the transcription, protein, metabolic, and polypeptide levels, to characterize the relationship between the different data and present an in-depth interpretation thereof. It ultimately informs a comprehensive understanding of biological systems [10, 11]. Multiomics can be used to acquire a deeper understanding of the flow changes from the group level to an integrated level, to explore how molecules and signaling pathways are affected, and to clarify the molecular mechanisms by which CRDs affect physical health. It also facilitates the use of precise intervention measures in the CRD population, thereby allowing for enhancements in their health management and improvements in their physical health. This study was designed as a prospective cohort study that would dynamically analyze multiomics data (e.g., proteomics, metabolomics, lipidomics, and polypeptidomics) from individuals with CRD to observe the effects of human physiology, psychology, and molecular multiomics on this patient population.

The primary aim of this study is to develop a precise health promotion model for the CRD population that considers multiomics data linked to lifestyle, clinical, and psychological data. The specific objectives for this cohort study include collecting a substantial amount of health multiomics data on individuals with CRD, drawing a dynamic molecular phenotype map of CRDs, as well as exploring the key molecules and signaling pathways that respond to CRDs.

## Methods/design

### Trial registration

The clinical study registration has been completed (Trial Registration No. ChiCTR2100047242,the URL:http://www.chictr.org.cn/edit.aspx?pid=127617&htm=4).

### Study design and setting

**Fig. 1:**
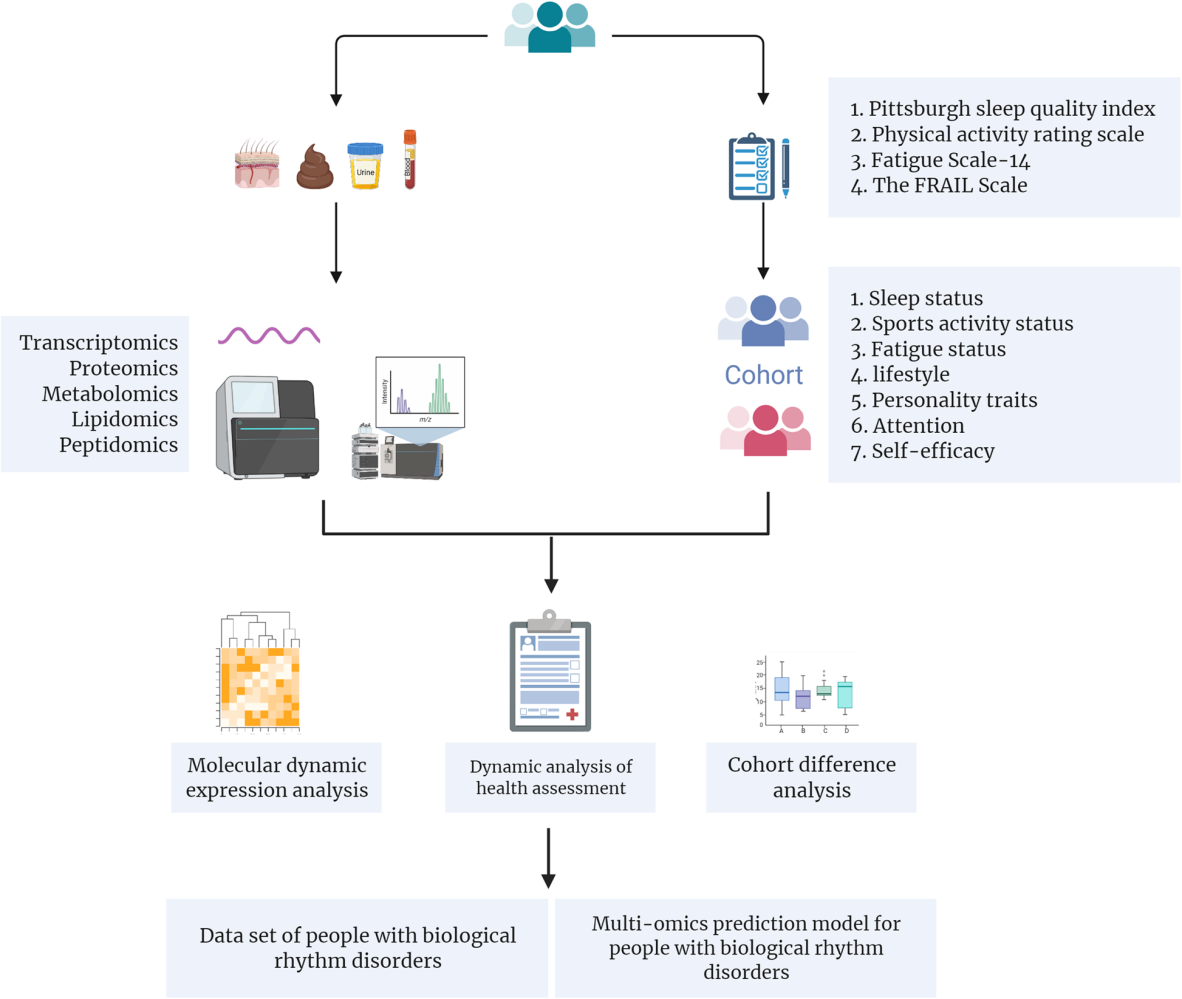
General overview of the study. Health questionnaire survey,physical examination and multiomics assays will be performed to collect data on the volunteers’ general health. Horizontal cohort (cohort difference analysis) and longitudinal cohort (dynamic analysis of health assessment, molecular dynamic expression analysis) data analysis will be performed to develop a precise health promotion model for the CRD population

This is a prospective, longitudinal cohort study. This study will begin in July 2022.Eligible volunteers with biological rhythm rules and biological rhythm disorders were recruited prospectively by key members of the research team who have been trained on inclusion/exclusion criteria and recruitment procedures. The participants will be visiting the hospital a total of 10 times for biological sample collection, lifestyle survey, and psychological evaluation every year. A general overview of the study is shown in Fig. 1.

The study is divided into three parts conducted over a 5-year period: First, a health questionnaire survey will be administered to collect data on the volunteers’ general characteristics, lifestyle, psychological assessment results, and follow-up health status (Table 1). Second, health examinations, including physical examination, laboratory examination, and electrocardiogram (ECG) activity monitoring, will be performed. The physical examination will mainly include measurements of body temperature, heart rate, respiration, systolic blood pressure, diastolic blood pressure, peripheral oxygen saturation, height, weight, and body fat. The laboratory examination will include evaluations of blood cells, biochemistry, renal liver function, cardiac function, thyroid function, as well as inflammatory markers. Dynamic ECG activity data will be obtained from the health smartwatch and portable dynamic ECG monitoring system that each volunteer will be asked to wear (Table 2). Third, multiomics assays, including transcriptomics, metabolomics, proteomics, polypeptidomics, and lipidomics data sets, will be performed by a professional company for the omics detection of biological specimens (i.e., blood, intestinal flora, urine, and saliva). The collection and processing of biological specimens will be completed by registered nurses and laboratory personnel.

**Table 1:** Health assessment of DMCRD

Classification	Measures
General information	sociodemographic characteristics, health status, job evaluation
Sleep status	Pittsburgh sleep quality index (PSQI)
Physical activity	physical activity scale (PARS-3)
Fatigue	Fatigue Assessment Scale (FAS)
Concentration ability	The CPT test
Weakness	FRAIL scale
Anxiety and Depression	Self-rating Anxiety Scale (SAS) Self-rating Depression Scale (SDS)
Dietary structure	Food frequency questionnaire (FFQ)
Nutrition	Nutrition risk screening (NRS 2002)

**Table 2:** Health examination of DMCRD

Classification	Measures
Physical examination	Body temperature, heart rate, respiration, systolic blood pressure, diastolic blood pressure, peripheral oxygen saturation, height, weight, BMI
Laboratory examination	Blood routine, biochemical routine, renal panel and liver function test, cardiac function, thyroid function, inflammatory markers, and immune markers (Cell T, Cell B)
Portable Health smartwatch	Dynamic ECG activity, Heart rate variability analysis
	Brain electrical activity
	Sleep quality, Physical activity

### Study population

Men and women aged between 18 and 40 years, with normal thinking and language expression ability, who have had a biological rhythm disorder for ≥3 months, and who could complete the physical examination as required and passed the physical examination were included in this study. Volunteers with any clinically diagnosed acute and chronic diseases, mental illness, or sleep disorders were excluded. Pregnant and lactating women were also excluded.

### Sample size

It is different to calculate the sample size of multiomics assays.100 volunteers who will be followed up for 5 years have been identified.

### Baseline and follow-up visits

The clinical study registration has been completed, and a study cycle of 5 years is currently planned. One hundred individuals with CRD were recruited and had undergone baseline health examination, health assessment, and biological specimen collection. A follow-up visit, during which health examination, questionnaire evaluation, and biological specimen collection will again be conducted, will be arranged every year (Table 3). The volunteers were added to a WeChat group, to which research information will be sent to ensure the significance of the sampling, thereby facilitating effective research communication and establishing the researchers’ accessibility. Members of our research team will regularly conduct one-to-one contact with the volunteers via WeChat or by telephone. The volunteers’ contact number and address will be updated regularly as well as sent to the researchers 1 week and 1 day before the next follow-up visit to prevent loss of contact.

**Table 3:** Details and procedures of the baseline study visit and follow-up periods

Follow-up period	Classification				
	Volunteer recruitment	General information	Health assessment	Health examination	multi-omics testing
Baseline	√	√	√	√	√
1 year	–	√	√	√	√
2 years	–	√	√	√	√
3 years	–	√	√	√	√
4 years	–	√	√	√	√
5 years	–	√	√	√	√

### Measurements and instruments

#### General information

As general attributes, the volunteers’ sociodemographic characteristics, such as age, sex, education, occupation, marital status, history of disease, and duration of biological rhythm disorder, were and at follow-up will be documented. In addition, their average working hours per week, shift frequency, night shift schedule and frequency, day shift schedule and frequency, as well as work intensity were and at follow-up will be recorded.

#### Health assessment

The health assessment included and at follow-up will include evaluations of the volunteers’ sleep, physical activity, fatigue, concentration ability, weakness, anxiety and depression, dietary structure, nutrition, and health status. The Pittsburgh Sleep Quality Index is used to investigate sleep status for the last month [12]. It includes 19 self-evaluation and 5 peer review items. In all, seven components are evaluated, namely, sleep quality, sleep time, sleep time phase, sleep efficiency, sleep disturbance, use of hypnotic drugs, and daytime dysfunction. The score for each component ranges from 0 to 3, with the total score ranging from 0 to 21. A higher score on the index indicates poorer sleep quality.

Physical activity was and at follow-up will be assessed using the Physical Activity Rating Scale. The scale specifically analyzes intensity, time, and frequency of exercise (amount of exercise = Intensity × Time × Frequency). The total score ranges from 0 to 100, with a score of ≤19 indicating a fluctuating amount of exercise, a score between 20 and 42 indicating a moderate amount of exercise, and a score of ≥43 indicating a large amount of exercise.

Fatigue was and at follow-up will be evaluated using the Fatigue Assessment Scale [13], which is a simple self-reporting tool for patients. The scale includes 10 judgment statements followed by 5 status ratings for each judgment statement, namely, “never” (1 point), “sometimes” (2 points), “frequently” (3 points), “often” (4 points), and “always” (5 points). The total fatigue, physical fatigue, and mental fatigue scores are counted according to each respondent’s status.

The time phase of persistent concentration, including visual, auditory, and audiovisual stimulus responses, was and at follow-up will be measured using a continuous performance test. Visual Basic will be used to develop a software that can automatically record the volunteers’ average response time, number of correct responses, and number of incorrect responses.

Weakness was and at follow-up will be assessed using the FRAIL scale [14], which analyzes fatigue, resistance, ambulation, illnesses, and loss of weight. The scale uses a yes/no format, with “yes” being equal to 1 point and “no” being equal to 0 points. The highest possible total score is 5 points, with a higher score indicating a higher possibility of weakness.

#### Health examination

Physical examination mainly included and at follow-up will include measurements of body temperature, heart rate, respiration, systolic blood pressure, diastolic blood pressure, peripheral oxygen saturation, height, weight, and body fat. Laboratory examination included and at follow-up will include evaluations of routine blood chemistry, routine biochemistry, renal liver function, cardiac function, thyroid function, and immune markers.

#### ECG monitoring

The volunteers will be fitted with a health smartwatch (PHOT W2-MTBD; Apple) and a portable dynamic ECG monitoring device on the day before their follow-up visit. The two devices will be used to monitor the subjects’ vital signs, ECG activities, brain electrical activity, sleep quality, and physical activity throughout the whole process. They will also be used to record the total cardiac beat, mean heart rate, long period (> 2 second), fastest heart rate, and slowest heart rate.

#### Specimen collection and processing

Blood, intestinal flora, urine, and saliva samples were and at follow-up will be collected from the volunteers before and after their day shift, before and after their night shift, as well as after a 24-hour shift. Venous blood samples (~ 15 mL) were and at follow-up will be taken and centrifuged for 20 minutes within 30 minutes. Intestinal flora, urine, and saliva specimens were and at follow-up will be collected in accordance with standard procedures. All specimens were and at follow-up will be transported to clinical laboratories within 4 hours and stored in a refrigerator at − 80 °C until use.

#### Multiomics analysis

Specimens were and at follow-up will be analyzed using a high-performance liquid chromatography/mass spectrometry system and tested multiple times. Nontargeted proteomics (260 proteomes), targeted proteomics (109 proteomes), nontargeted metabolomics (728 metabolites), semi-targeted lipidomics (710 lipids), transcriptomics (16,000 RNA molecules), and other multiomics data were and at follow-up will be evaluated.

### Data analysis

Data on work, sleep, physical activity, fatigue, and concentration, among others, will be studied using horizontal cohort data analysis by group. Continuous variables with a normal distribution will be compared using *t* test or analysis of variance, whereas those with a non-normal distribution will be analyzed using a nonparametric test. Categorical variables will be analyzed using the *χ*^2^ test. The molecular expression of different horizontal queues will be compared using a computer.

Longitudinal cohort data analysis will be performed according to multipoint time order. Cluster analysis of longitudinal trajectories based on multiple sets of scientific data will be used. The machine learning method of binomial classification will be adopted to construct an accurate health prediction model for individuals with CRD based on multiomics data. Kolmogorov-Smirnov, F1, and receiver operating characteristic curves will be used to test the prediction sensitivity, specificity, and accuracy of the model.

## Discussion

Our work will describe the molecular mechanisms of circadian rhythm disturbance in detail. Individuals with CRD were included in the study. A series of long-term follow-up will be implemented to collect the volunteers’ health status and to ultimately generate a set of multiomics data. The characteristics of our research methods differ from those of research methods used in previous studies. Using multiomics analysis will help us not only explore the relationship between CRDs and the health of the molecules and signaling pathways in the body but also identify the molecular mechanisms by which CRDs affect physical health as well as detect potential clinical biomarkers and therapeutic targets. This research aims to establish an accurate health prediction model for individuals with CRD through multiple sets of scientific data and health assessments.

Our research design has three main characteristics: First, this is a prospective cohort study focusing on the health management of individuals with CRD. The various health assessment data and multiple sets of scientific data obtained in this study can be used as evidence to support the clinical application value of precision medicine. Second, this is a long-term cohort study. Data will be collected through multiple follow-up visits to assess the causal relationship and molecular mechanisms between circadian rhythm and sleep disorders, weakness, as well as illness. Third, this study will establish an accurate health prediction model based on multiomics analysis. Previous single-group studies have only revealed molecular data related to circadian rhythm disturbance at a single level. As this study will be using multiomics analysis, it can produce comprehensive and in-depth results.

This study will analyze the impact of CRDs on health status through health assessment and multiomics data to provide key evidence and an accurate prediction model for the health management of the CRD population. In addition, our data can serve as the basis for further routine health management and clinical disease research. At present, 100 volunteers who will be followed up for 5 years have been identified. However, we hope to increase the sample size and extend the follow-up period if our research resources would allow us to do so.

### Trial status

Protocol version: v1.1, 01/01/2022.

The study began recruitment on 01/07/2022. We plan to complete recruitment by 31/12/2022.

## Data Availability

The dateset will available from ResMan, DOI: http://www.medresman.org.cn.

## Abbreviation

CRDs: Circadian rhythm disorders

## References

[1] Reppert SM, Weaver DR (2002). Coordination of circadian timing in mammals. Nature.

[2] Irwin MR (2015). Why sleep is important for health: a psychoneuroimmunology perspective. Annu Rev Psychol.

[3] Abbott SM, Malkani RG, Zee PC (2020). Circadian disruption and human health: a bidirectional relationship. Eur J Neurosci.

[4] Ju S-Y, Choi W-S (2013). Sleep duration and metabolic syndrome in adult populations: a meta-analysis of observational studies. Nutr Diabetes.

[5] Brown DL, Feskanich D, Sanchez BN, Rexrode KM, Schernhammer ES, Lisabeth LD (2009). Rotating night shift work and the risk of ischemic stroke. Am J Epidemiol.

[6] Forman JP, Curhan GC, Schernhammer ES (2010). Urinary melatonin and risk of incident hypertension among young women. J Hypertens.

[7] Straif K, Baan R, Grosse Y, Secretan B, El Ghissassi F, Bouvard V, Cogliano V (2007). Carcinogenicity of shift-work, painting, and fire-fighting. Lancet Oncol.

[8] Koritala BSC, Porter KI, Arshad OA, McDermott JE, Gaddameedhi S (2021). Night shift schedule cause circadian dysregulation of DNA repair genes and elevated DNA damage in human. J Pineal Res.

[9] Vaccaro A, Kaplan Dor Y, Nambara K, Rogulja D (2020). Sleep loss can cause death through accumulation of reactive oxygen species in the gut. Cell.

[10] Karcewski KJ, Smyder MP (2018). Integrative omics for health and disease. Nat Rev Genet.

[11] Ritchie MD, Holzinger ER, Li R (2015). Methods of integrating data to uncover genotype-phenotype interactions. Nat Rev Genet.

[12] Buysse DJ, Reynolds CF, Monk TH (1989). The Pittsburgh sleep quality index:a new instrument for psychiatric practice andresearch. Psychiatry Res.

[13] De Vries J, Michielsen H, Van Heck GL, Drent M (2004). Measuring fatigue in sarcoidosis: the fatigue assessment scale (FAS). Br J Health Psycho.

[14] Morley JE, Malmstrom TK, Miller DK (2012). A simple frailty questionnaire (FRAIL) predicts outcomes in middle aged African Americans. J Nutr Health Aging.

